# Environmental and resource burdens associated with world biofuel production out to 2050: footprint components from carbon emissions and land use to waste arisings and water consumption

**DOI:** 10.1111/gcbb.12300

**Published:** 2016-01-05

**Authors:** Geoffrey P. Hammond, Bo Li

**Affiliations:** ^1^ Department of Mechanical EngineeringUniversity of BathBathBA2 7AYUK; ^2^ Institute for Sustainable Energy and the Environment (ISEE)University of BathBathBA2 7AYUK

**Keywords:** carbon emissions, embodied energy, energy–land–water nexus, environmental footprints, land use, sustainability, transport, waste arisings, water consumption, world biofuel production

## Abstract

Environmental or ‘ecological’ footprints have been widely used in recent years as indicators of resource consumption and waste absorption presented in terms of biologically productive land area [in global hectares (gha)] required *per capita* with prevailing technology. In contrast, ‘carbon footprints’ are the amount of carbon (or carbon dioxide equivalent) emissions for such activities in units of mass or weight (like kilograms per functional unit), but can be translated into a component of the environmental footprint (on a gha basis). The carbon and environmental footprints associated with the world production of liquid biofuels have been computed for the period 2010–2050. Estimates of future global biofuel production were adopted from the 2011 *International Energy Agency* (IEA) ‘technology roadmap’ for transport biofuels. This suggests that, although *first generation biofuels* will dominate the market up to 2020, advanced or *second generation biofuels* might constitute some 75% of biofuel production by 2050. The overall environmental footprint was estimated to be 0.29 billion (bn) gha in 2010 and is likely to grow to around 2.57 bn gha by 2050. It was then disaggregated into various components: bioproductive land, built land, carbon emissions, embodied energy, materials and waste, transport, and water consumption. This component‐based approach has enabled the examination of the *Manufactured* and *Natural Capital* elements of the ‘four capitals’ model of sustainability quite broadly, along with specific issues (such as the linkages associated with the so‐called energy–land–water nexus). Bioproductive land use was found to exhibit the largest footprint component (a 48% share in 2050), followed by the carbon footprint (23%), embodied energy (16%), and then the water footprint (9%). Footprint components related to built land, transport and waste arisings were all found to account for an insignificant proportion to the overall environmental footprint, together amounting to only about 2%

## Nomenclature

**Abbreviations**
CO_2_: carbon dioxide
CO_2e_: carbon dioxide equivalent
EFA: environmental (or ecological) footprint analysis
EU: European Union
FAO: Food and Agricultural Organization of the United Nations
FGB: first generation biofuels
GHG: ‘greenhouse’ gas
IEA: International Energy Agency
iLUC: indirect land‐use change
LCA: environmental life cycle assessment
LUC: (direct) land‐use change
OECD: Organisation of Economic Co‐operation and Development
SGB: second generation biofuels
SRC: short rotation coppice

**Symbols**
aa_*i*_: each major category of consumption
*c_f_*: carbon footprint *per litre of biofuel* (gha or tC)
*c*_*i*_: annual consumption of an item
*C*_W_: carbon weight *per litre of biofuel* (tC)
*e_f_*: environmental footprint *per capita* (gha)
EF: total environmental footprint (gha)
*N*: population size
*p*_i_: average annual yield of an item

**Subscript**
*i*: *i*th category of consumption

## Introduction

### Background

Humans were almost wholly dependent on finite fossil and nuclear fuels for energy resources at the turn of the Millennium; amounting to about 77% and 7% of global primary energy needs, respectively (Everett *et al*., 2012). ‘Traditional’ renewable energy sources, such as burning fuelwood and dung or using water and windmills, accounted for 11% of these worldwide requirements. Large‐scale hydroelectric power contributed 3%, and other renewables (including modern wind turbines and liquid biofuels) contributed just 2%. Sustainable development in a strict sense requires a reversal of these roles (Hammond, 2000; Hammond & Jones, 2011), but it is unlikely that renewable energy technologies could meet a high proportion of industrial countries’ energy demand before at least the middle of the 21st century. This is partly due to the conflict between the needs of environmental sustainability and the downward economic pressures on energy prices arising from moves towards energy market liberalization, as well as the post‐2008 economic recession in the industrialized world. The European Union (EU) target of 20% renewables use by the year 2020 (with 10% of ‘green fuels’, principally biofuels, for transport) was seen by many analysts as being overly ambitious. Nevertheless, substantial progress has been made across much of Europe in terms of its ‘20‐20‐20’ policy framework (EREC, 2013). This reflects a binding target of a 20% reduction in ‘greenhouse’ gas (GHG) emissions by 2020 from 1990 levels (against a longer term target of an 80% fall by 2050), increasing the amount of energy produced from renewable resources to a binding level of 20% by 2020 and a nonbinding aim of a 20% improvement in the EU energy efficiency over the same timescale.

Transport underpins the mobility of people around the world, and it presently accounts for around 20% of global anthropogenic carbon dioxide (CO_2_) emissions (RoySoc, 2008; Hammond *et al*., 2012): an unwanted side effect. The adoption of liquid biofuels in the transport sector has therefore been seen, particularly by the EU (Hammond *et al*., 2008; EREC, 2013), as a means for meeting climate change mitigation targets, enhancing regional energy or fuel security and contributing to rural development (through the provision of an alternative source of income in otherwise depressed agricultural communities). Biomass can be converted into premium‐quality liquid biofuels and biochemicals (Tester *et al*., 2005; Hammond & Seth, 2013). [A narrative description of various biofuels and their feedstocks, along with a discussion of the impact of so‐called upstream emissions (those emanating upstream of the biofuel use, typically in an internal combustion engine) can be found in the Supporting information.] But the deployment of biofuels has been linked to significant adverse impacts in terms of direct and indirect land‐use change (LUC and iLUC), loss of biodiversity and ecosystem services (Elghali *et al*., 2007; Hammond *et al*., 2008) and competition with food production. First generation biofuels (FGB), for example, are produced primarily from food crops (Hammond *et al*., 2012) and are limited by their inability to achieve targets for oil‐product substitution (without threatening food supplies and biodiversity) and for GHG reductions. In contrast, more advanced or second generation biofuels (SGB) are generally produced from agricultural or crop ‘wastes’ (such as straw) and from nonfood energy crops, which significantly reduces these negative impacts (Hammond *et al*., 2012). Potential feedstocks and conversion routes (Hammond *et al*., 2008) therefore need to be assessed against the full range of sustainability considerations and over the full life cycle of the biofuel supply chain (Elghali *et al*., 2007; RoySoc, 2008; Hammond & Jones, 2011; Hammond *et al*., 2012): from ‘field‐to‐(‘*gas*’ or petrol station) forecourt’ or ‘seed‐to‐wheel’. Only in this way will the true consequences of a given biofuel – environmental, economic and social – be determined (Hammond & Jones, 2011).

### Biofuels, water consumption and the ‘energy–land–water nexus’

Water resources and their footprints consist of three elements: so‐called *blue*,* green* and *grey* water (see, for example, Mekonnen & Hoekstra, 2011). ‘*Blue*’ water is associated with the volume of freshwater that evaporates from the global blue water resources (surface water and ground water) to produce the goods and services consumed by the individual or community. In contrast, ‘*green*’ water is the volume of water evaporated from the global green water resources (rainwater stored in the soil as soil moisture). Finally, ‘*grey*’ water is the volume of polluted water that associates with the production of all goods and services for the individual or community. This can be estimated as the volume of water that is required to dilute pollutants to such an extent that the quality of the water remains at or above agreed water quality standards.

The term ‘*Natural Capital*’ (Costanza & Daly, 1992; Ekins, 1992; Aronson *et al*., 2006; Turner & Daily, 2008; Daly & Farley, 2011) is typically used to denote the biotic or abiotic stocks and flows that yield natural assets and tangible natural resources. These in turn provide *ecosystem services* (ES), or ‘living natural capital’ (Turner & Daily, 2008), such as those required for food (including those associated with the pollination in crops), timber and the absorption or recycling of human waste arisings (including CO_2_), as well as water catchment and erosion control. Maintenance of this *Natural Capital* is consequently central to securing environmental security and sustainability over the longer term. A key subset is the so‐called nexus, or set of complex interactions, between energy requirements, land uses and water consumption levels worldwide (Liu *et al*., 2015). This energy–land–water [ELW] nexus (Brandi *et al*., 2013) gives rise to multiple positive and negative impacts that have recently been widely debated in policymaking circles. Energy generation is obviously the main driver for anthropogenic climate change, whilst there are competing demands on land use [both LUC and iLUC (Hammond & Jones, 2011; Hammond & Seth, 2013)] for both food and biofuel production. Water is needed for drinking, irrigation, food and biofuel crop production, hydro‐electric dams and various leisure pursuits. They are all exacerbated by increasing ELW demands arising from the growth in world population that is moving towards 8 billion (bn) in 2025 and 9.5 bn by 2050 (Cranston & Hammond, 2010), as well as human socio‐economic developments generally. Such demands are often framed in terms of energy, food or water ‘security’. It is argued that a strategy which focuses on just one element of the nexus, without considering the others, is likely to lead to major unintended consequences. Thus, many have advocated the need for an integrated approach to the management and governance of nexus issues across various sectors and at different scales to ensure sustainability. This would necessitate research and the modelling of ELW impacts within an informed, transparent and integrated framework for planning and decision support.

### The issues considered

Environmental or ‘ecological’ footprints (*e_f_*) have been widely used in recent years as indicators of resource consumption and waste absorption transformed on the basis of biologically productive land area [in global hectares (gha)] required *per capita* with prevailing technology (Chambers *et al*., 2000; Hammond, 2006; Eaton *et al*., 2007; Cranston & Hammond, 2010; Alderson *et al*., 2012). They represent a partial measure of the extent to which an activity [that might be associated with communities, technologies, or systems] is ‘sustainable’ (Eaton *et al*., 2007; Cranston & Hammond, 2010). In contrast, ‘carbon footprints’ (*c_f_*) are the amount of carbon [or ‘carbon dioxide equivalent’ (CO_2e_)] emissions associated with such activities (Alderson *et al*., 2012; Cranston & Hammond, 2012). But, unlike environmental footprints, they are generally presented in terms of units of mass or weight (kilograms per functional unit), rather than in spatial units (such as gha). These carbon footprints have become the ‘currency’ of debate in a climate‐constrained world (Cranston & Hammond, 2010). Such footprints are increasingly popular ecological indicators, adopted by individuals, businesses, governments and the media alike. For this study, *e_f_* was therefore broken down, respectively, into various components: carbon emissions (effectively *c_f_*), embodied energy, transport, built land, water and waste. This component‐based approach was then employed to calculate *e_f_* on an annual basis from 2010 to 2050 using projections of world biofuel production published by the *International Energy Agency* (IEA) as part of their ‘technology roadmap’ for transport biofuels (IEA, 2011). It facilitates the examination of the *Manufactured* and *Natural Capital* elements of what was originally known as the ‘four capitals’ model of sustainability (Ekins, 1992), along with specific issues [such as the linkages associated with the so‐called ELW nexus (Brandi *et al*., 2013)]. This approach provides a means of comparing the various footprint components on a common basis. This is not without potential controversy, but yields a better way of comparing environmental sustainability topics than many of the alternatives.

Hammond & Seth (2013) applied similar footprint methods to determine the environmental and resource burdens arising from the global production of liquid biofuels up until about 2020. They adopted production estimates reported by the OECD‐FAO (2010) for the period 2007–2019, when FGB are likely to be dominant. [Comparisons of the present results with those of the earlier study of global biofuel footprints to 2019 by Hammond & Seth (2013) can be found in the Supporting information (see, for example, Fig. S1–S3).] Recently, Liu *et al*. (2015) cited the biofuels footprint study of Hammond & Seth (2013) as an example of the employment a ‘systems integration framework’ for global sustainability assessment. The present results utilize the projections developed by the IEA as part of their technology roadmap for transport biofuels (IEA, 2011; see Table 1). These extend out to 2050 and therefore account for the growing impact of SGB. In addition to assessing the carbon and environmental footprints associated with the IEA transport biofuel projections, the opportunity has been taken to critically reappraise the detailed way in which the individual footprint components have been evaluated. In particular, the water footprint of liquid biofuels has been determined using the recent work of Hoekstra and his co‐workers (see, for example, Mekonnen & Hoekstra, 2011). That has enabled a cross‐comparison of methods for calculating the environmental footprint components and thereby helping to better determine the relative shares of the different biofuel components out to 2050, including that associated with water consumption.

**Table 1:** Global biofuel demand out to 2050

Year	Conventional bioethanol	Bioethanol cane	Bioethanol SRC	Conventional biodiesel	Advanced biodiesel	Biojet	Biomethane	Total
	Biofuel demand (EJ)							
2010	1.29	0.44	0.00	0.53	0.00	0.00	0.00	2
2015	1.35	0.90	0.15	0.68	0.15	0.08	0.00	3
2020	1.50	1.44	0.45	0.90	0.38	0.15	0.23	5
2025	1.20	1.88	1.05	0.98	1.13	0.83	0.38	7
2030	0.98	2.11	1.88	0.90	1.96	1.35	0.98	10
2035	0.45	2.48	2.56	0.60	3.61	2.41	1.28	13
2040	0.15	2.63	3.46	0.23	5.34	3.16	1.66	17
2045	0.08	2.86	4.14	0.08	7.98	5.04	3.76	24
2050	0.00	3.24	5.04	0.00	10.91	6.70	5.87	32

*Source*: IEA (2011).

## Materials and methods

### The IEA technology roadmap on transport biofuels

The IEA ‘technology roadmap’ on transport biofuels (IEA, 2011) suggests that, although FGB will dominate the market up to 2020 [(in line with the OECD‐FAO projections analysed by Hammond & Seth (2013)], SGB might constitute some 75% of biofuels production by 2050. They argue (IEA, 2011) that the amount of global biofuels for transport could rise nearly sevenfold over the period 2020–2050 [to just over 30 ExaJoules (EJ) equivalent primary energy demand *per annum*]: see again Table 1. That would represent some 27% of global transport fuel supply by the middle of the 21st century in contrast to only about 2% today (Fairley, 2011). Such biofuel demands fall within the ‘low band estimates’ according to the classification of Slade *et al*. (2011) in their comprehensive global bioenergy resource assessment. A recent review by Searle & Malins (2014) suggested that the maximum availability of global biofuels was about 20 EJ year^−1^, although they argued that this was ‘similar’ to the global demand predicted by the IEA (2011), that is ~30 EJ year^−1^.

The International Energy Agency assumed that the growth of the world economy over the longer term (period from 2008 to 2035) will slow down in OECD and non‐OECD regions (IEA, 2011). On the other hand, they suggest that global population is likely to more than double against the 1950 level, increasing from 7 bn in 2011 to around 9.5 bn by 2050 (see also Cranston & Hammond, 2010; Alexandratos & Bruinsma, 2012). The projections of global biofuel production out to 2050 (IEA, 2011) were based on the IEA ‘BLUE Map Scenario’ adopted for their energy technology perspectives study (IEA, 2010a). This employed a ‘deep’ GHG emission reduction target of 50% energy‐related CO_2_ emissions by 2050 (against a baseline of 2005). Global biofuel demand was then estimated to increase from 55 million tonnes of oil equivalent (Mtoe) to 750 Mtoe in 2050. This implies that the world share of biofuel in total transport fuel demand would increase from 2% to 27% by 2050 (IEA, 2011). These bioethanol projections indicated that conventional bioethanol from sugar beet and corn would begin to grow slowly from 2015, although it would be replaced rather more rapidly by advanced bioethanol production from sugarcane and cellulosic feedstock after about 2020. Biodiesel produced from edible vegetable oil was assumed by the IEA (2011) to be the most likely route to biodiesel production during the 2010–2020 period, but some novel biodiesel technologies might help meet biofuel demands after the year 2020. In line with IEA roadmap projections, growth in advanced biofuel demand overall would reach 10 EJ year^−1^ by 2030 with a further three times increase of SGB production by 2050 (IEA, 2011) to about 32 EJ year^−1^ (see Table 1).

The growth requirement of biomass feedstock as the result of increasing biofuel production in accordance with the IEA roadmap could potentially lead to competition between food crop production on arable land and that for biofuels. The total requirement of bioenergy expected under the BLUE Map Scenario (IEA, 2010b) was around 145 EJ in 2050, of which 65 EJ was for biofuels and 80 EJ is for heat and power generation. Almost 50% of future biofuels and biomethane was then assumed to be produced via advanced technologies, such as bioethanol derived from short rotation coppice (SRC), residues and other waste materials (IEA, 2011). Global biofuel demand, which requires 2% of world arable cropland today, will increase to around 6% in 2050. This corresponds to a growth from the present 30 to 100 Mha in 2050, involving cropland, pastures and some marginal land (IEA, 2011). According to *Food and Agricultural Organization* (FAO) projections (Alexandratos & Bruinsma, 2012), an additional 70 Mha arable land expansion would be expected to meet the global population growth by 2050, which involves an expansion in developing countries (such as sub‐Saharan Africa and Latin America) of some by 120 Mha, and a fall of 50 Mha in developed countries (Alexandratos & Bruinsma, 2012). The potential conflict between the requirements of world cereal, sugar and vegetable oil production may be minimized by the utilizing marginal or idle land and forest land, as well as encouraging rapidly the adoption and development of advanced biofuel technologies (such as biofuels produced from cellulosic, residues and wastes). The take‐up of the latter is heavily reliant on innovation policies, incentives and regulations supported by national governments (Hammond & Seth, 2013).

### Carbon and environmental footprinting

#### The environmental footprint methodology

The use of ‘ecological’ or environmental footprint analysis (EFA) has grown in popularity over the last couple of decades, both in Europe and North America. They provide a simple, but often graphic, measure of the environmental impact of human activity: whether or not in the foreseeable future humanity will be able to ‘tread softly on the Earth’ (Hammond, 2000). William Rees used footprint analysis in its basic form to teach planning students for some 20 years [see Wackernagel & Rees (1996)]. He decided to adopt the term ‘ecological footprint’ in the early 1990s, rather than ‘appropriated carrying capacity’ that he had previously used, after buying a new television set (Hammond, 2007). It had a smaller footprint (that is, took up less space) than his old model. The terms ‘environmental’ and ‘ecological’ footprints are used interchangeably here [as they were previously by Hammond (2006), Eaton *et al*. (2007), Cranston & Hammond (2010), Alderson *et al*. (2012) and Hammond & Seth (2013)], although the former expression is preferred. Ecology is that branch of biology dealing with the interaction of organisms and their surroundings. ‘Human ecology’, sometimes used for the study of humans and their environment, is closer to the usage implied by footprint analysis.

Footprint calculations involve several steps. Initially, the land area per functional unit (e.g. *per capita* or, in the present case, per kg or tonne of biofuel) appropriated for each major category of consumption (aa_*i*_) is determined:

$${\text{aa}}_{i}=\frac{c_{i}}{p_{i}}∼\frac{\text{Annual consumption of an item}}{\text{Average annual yield}}.$$

**Figure 1:**
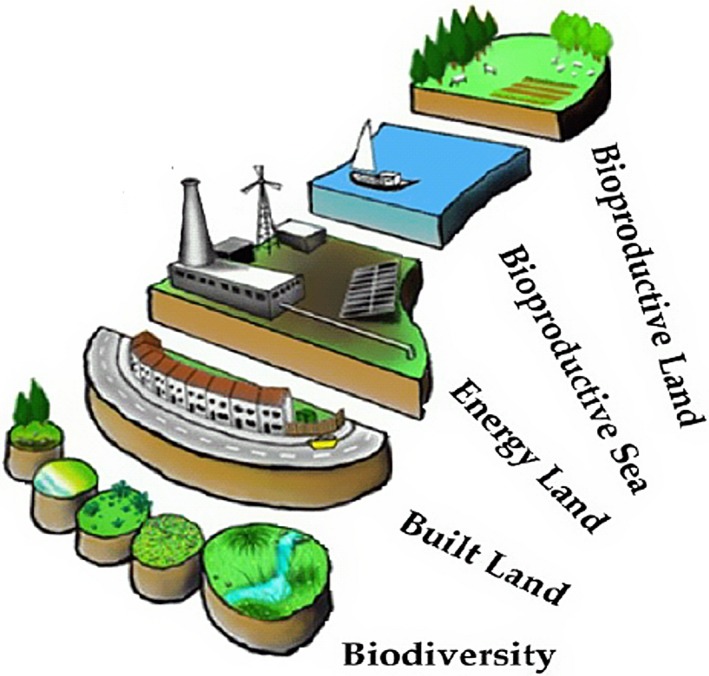
Schematic representation of the environmental footprint and its land types [*Source*: adapted from Eaton *et al*. (2007), adapted from Chambers *et al*. (2000)].

**Figure 2:**
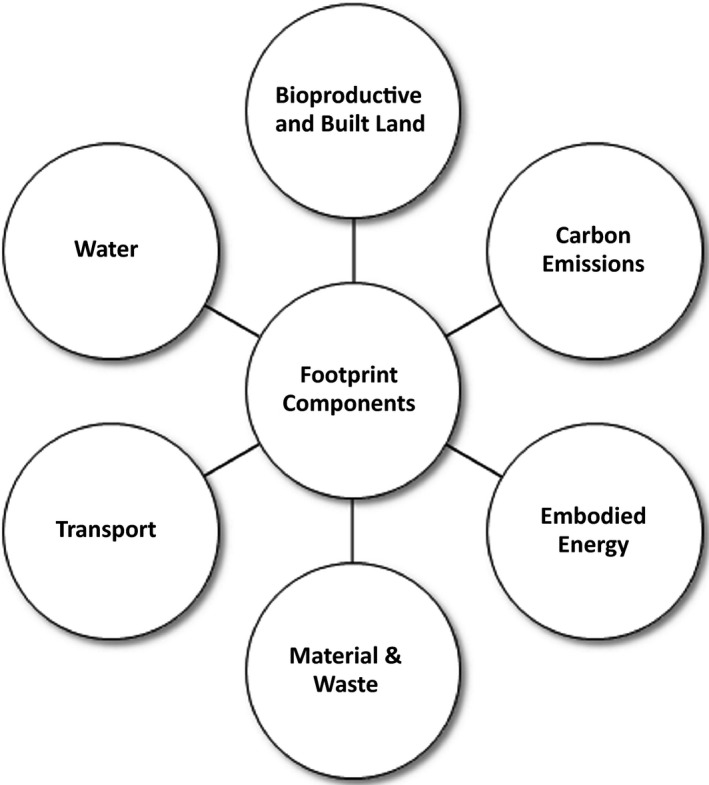
Schematic representation of the component‐based approach to environmental footprint analysis [*Source*: adapted from Eaton *et al*. (2007), based on the method developed by Simmons *et al*. (2000)].

In the original version of EFA employed by Wackernagel & Rees (1996), four consumption categories were identified: energy use, the built environment (the land area covered by a settlement and its connection infrastructure), food and forestry products. This is a restricted subset of all goods and services consumed which was determined by the practical requirements of data gathering and influenced by the development of the technique in a Canadian setting. Five land types have typically been employed: Chambers *et al*. (2000), for example, adopted bioproductive land, bioproductive sea, energy land, built land and the land needed to secure biodiversity as their categories (see Fig. 1). The six components subsequently analysed in the comparative study of urban and rural communities by Eaton *et al*. (2007), in addition to the carbon footprint, were ‘built land’, ‘embodied energy’, ‘materials and wastes’, ‘transport’ and ‘water’ (see, for example, Fig. 2). To calculate the footprint per functional unit (*e_f_*) in global hectares (gha), the appropriated land area for each consumption category is then summed to yield, after Wackernagel & Rees (1996):

$$e_{f}\;=\sum_{i=1}^{i=n}{\text{aa}}_{i}.$$

One global hectare represents a hectare (ha) of biologically productive land at the average global productivity. The different footprint components (such as those depicted in Fig. 2) need to be normalized, so that global hectares account for disparities in land productivities. This computation then leads to a matrix of consumption categories and land‐use requirements, which is ideally suited to a spreadsheet implementation. To determine the total footprint (EF) for a given activity (e.g. community, product or resource), the functional unit figure (*e_f_*) is simply multiplied by the relevant population size (N), thus [following Wackernagel & Rees (1996)]:

$$\text{EF =}\;e_{f}\left(N\right).$$

Methods for calculating the environmental and carbon footprints of the world biofuel production have been employed based on historic data and projections out to 2050. This footprint analysis is consistent with that developed by the *Global Footprint Network* (GFN) [http://www.footprintnetwork.org/] and related bodies. Hammond & Seth (2013) estimated the effect of the uncertainties in the constituent data related in their recent biofuel footprint study using an established procedure for uncertainty analysis [as previously adopted by Eaton *et al*. (2007) and Alderson *et al*. (2012)], although that for the total environmental footprint was found to be only about ±3%. However, they noted that the OCED‐FAO global biofuel projections employed in their study were deterministic in nature, rather than stochastic. Consequently, the scatter in the footprint calculations was principally dependent on the variation in the estimates of year‐on‐year global biofuel projection into the future. Similar reasoning applies to uncertainties emanating from the IEA projection used here, and therefore, the uncertainties have not been explicitly determined (although these would be around ±3%, as in the case of the study by Hammond & Seth, 2013).

#### The carbon footprint component

The concept of the ‘carbon footprint’ (cf) is rooted within the framework used to determine the eco‐footprint. However, Hammond (2007) noted that a ‘footprint’ would normally be measured in spatial units [such as global hectares (gha)], but that the carbon footprint is typically presented in mass (or weight) units, that is kilograms (kg) or tonnes (t). He therefore argued that it should perhaps be termed a ‘carbon weight’ (*C*_W_) or something similar. Wiedmann & Minx (2008) reviewed various suggestions, including that of Hammond (2007), and then proposed a definition for the ‘carbon footprint’ as including the ‘total amount of CO_2_ emissions that is directly and indirectly caused by an activity’. Unfortunately, no definition has been formally adopted in a ‘standard’ with the agreement of the communities involved. Indeed, many organizations have adopted the use of the term carbon footprint when assessing the CO_2_ emissions released during various processes or activities, although these are again measured in tonnes of CO_2_ (Hammond, 2007; Wiedmann & Minx, 2008).

#### Other components of the environmental footprint

The initial phase of footprint analysis involves the collection of consumption data covering the various components (Chambers *et al*., 2000; Simmons *et al*., 2000; Eaton *et al*., 2007). This yields the flow of resources into and out of the global biofuel production sector. Proxy (or secondary) data adapted from international statistics were employed in the absence of sector‐specific obtained (or primary) data (Eaton *et al*., 2007; Alderson *et al*., 2012). This collation and analysis of data is highly disaggregated with many individual items of information. In addition to the consumption data needed for footprint analysis, yield and conversion (or ‘equivalence’) factors were required. Equivalence factors are a productivity‐based scaling parameter (Wackernagel & Rees, 1996; Chambers *et al*., 2000) that converts a specific land type (e.g. cropland, pasture, forest pasture, forest or fishing ground) into a universal unit of bioproductive land area (in gha). In the case of land types (e.g. arable or cropland) with productivity higher than the average productivity of all bioproductive land and water on the planet, the equivalence factor is > 1. According to Alderson *et al*. (2012), primary cropland has an equivalence factor of 2.10 (see also Hammond & Seth, 2013). Thus, to convert an average ha of cropland to the equivalent gha, it is multiplied by this cropland equivalence factor. In contrast, grazing land has a lower productivity than cropland (~0.47). More recent figures for equivalence factors, albeit slightly different from those used in the present studies, are tabulated online by the GFN.

The EFA resource components had to be identified and categorized to reflect broad and identifiable policymaking categories, which match the consumption of ‘natural capital’ (Eaton *et al*., 2007; Cranston & Hammond, 2010). In this study, these components were as follows (Simmons *et al*., 2000; Eaton *et al*., 2007):

- Bioproductive and Built Land: Land appropriated for biofuels development.
- Embodied Energy: The quantity of energy required for the processing equipment or to process fuels for the sector (Hammond & Jones, 2008).
- Materials and Waste: Consumption of products and materials for biofuels development and associated waste arisings.
- Transport: ‘Full fuel cycle’ transportation requirements.
- Water: The use of water for biofuels development.

‘Double accounting’ can arise when the embodied energy component (Hammond & Jones, 2008) includes the ‘process energy’ used in production; fuels for fertilizer production here. Thus, in this study, the embodied energy incorporates only the ‘upstream’ use of energy, whilst the carbon footprint represents the direct fuel inputs for biofuels development. This practice was first adopted by Alderson *et al*. (2012).

### Determination of the biofuel footprint components

#### Bioproductive land

‘Bioproductive land’ consists of arable land, forests and pasture, as well as (where appropriate) bioproductive sea (Chambers *et al*., 2000). The productivity of each land type will vary, but they will normally yield significant animal and plant output (Chambers *et al*., 2000). Consequently, the bioproductive land component of the environmental footprint calculated here included the land required for the cultivation of the different feedstocks that produce biofuels. The footprint component per litre for the IEA estimates of global biofuel production (IEA, 2011) was therefore computed as follows (Alderson *et al*., 2012):

$$\begin{array}{cc} & \text{Bioproductive land footprint component}\\ & \text{(gha per litre of biofuel)}\\ & =\text{Area of productive land (ha per litre of biofuel)}\\ & \;\times \text{Conversion factor}\;(\text{gha}\;{\text{ha}}^{-1}) \end{array}$$

$$\begin{array}{cc} & \;\text{Conversion factor}\;(\text{gha}\;{\text{ha}}^{-1})\\ & \;=\text{Global crop yield factor}\times \text{Equivalence factor}\;(\text{gha}\;{\text{ha}}^{-1}) \end{array}$$

A global crop ‘yield factor’ of 2.44 [as suggested by Alderson *et al*. (2012)] and the related equivalence factor of 2.1 were employed (following Hammond & Seth, 2013) to evaluate the amount of bioproductive land required per litre of biofuel. As a result, a conversion factor of 5.124 gha ha^−1^ for the bioproductive land component was obtained (Hammond & Seth, 2013).

#### Built land

Built land is land whose productive capacity has been largely utilized (or ‘lost’) for development purposes (Chambers *et al*., 2000), that is for buildings, roads and the like. In this study, the built land footprint component is represented by the land occupied for the construction of biorefineries and the associated infrastructure. The footprint component per litre of biofuel for the IEA global biofuel projections (IEA, 2011) was computed as follows (Alderson *et al*., 2012):

$$\begin{array}{cc} & \text{Built land footprint component (gha per litre of biofuel)}\\ & \;=\text{Area of developed land (ha per litre of biofuel)}\\ & \;\times \text{Conversion factor}\;(\text{gha}\;{\text{ha}}^{-1}) \end{array}$$

$$\begin{array}{cc} & \;\text{Conversion factor}\;(\text{gha}\;{\text{ha}}^{-1})\\ & \;=\text{Global crop yield factor}\times \text{Equivalence factor}\;(\text{gha}\;{\text{ha}}^{-1}) \end{array}$$

The quantity of built land required to produce a unit litre of biofuel was estimated based on the assumption that the biofuel refineries and associated infrastructure were built onsite on crop land. Consequently, the potential crop land that has been replaced effectively represents the built land. To adjust the built land for its relative productivity, the global crop yield factor of 2.44 (Hammond & Seth, 2013) was used, and hence, a related equivalence factor of 2.1 gha ha^−1^ was employed. The resulting conversion factor was once again taken as 5.124 gha ha^−1^ of crop land. Finally, this value was then multiplied by the IEA biofuel projections (IEA, 2011) to estimate the built land component. Simmons *et al*. (2000) adopted an equivalence factor of 2.82 gha ha^−1^ for what they termed built‐up area, which was subsequently used by Chambers *et al*. (2000).

#### Carbon emissions

The carbon component of the footprint was calculated using ‘carbon weight’ (*C*_W_) values and represents the amount of land required to sequester carbon. The carbon weight is the amount of carbon released in tonnes per tonne of biofuel produced by each global biofuel plant, and then burnt to yield the final energy service [e.g. in a vehicle internal combustion (IC) engine]. Therefore, the global carbon footprint per litre of biofuel from each type was calculated in a similar manner to Hammond & Seth (2013); following Alderson *et al*. (2012):

$$\begin{array}{cc} & \text{Carbon footprint component (gha per litre of biofuel)}\\ & \;=\text{Carbon weight (tC per litre of biofuel)}\\ & \;\times \text{Conversion factor (gha per tC)} \end{array}$$

$$\begin{array}{cc} & \;\text{Conversion factor (gha per tC)}\\ & \;=\frac{\text{Carbon responsibility}\times \text{Equivalence factor}\;(\text{gha}\;{\text{ha}}^{-1})}{\text{World carbon absorption factor}\;(\text{tC}\;{\text{ha}}^{-1})} \end{array}$$

Carbon sequestration by the global biological system through biological processes influences the world carbon cycle. The primary natural carbon absorber is forest land, which accounted for 69% of overall carbon sequesters. This is termed the ‘Carbon Responsibility’ in the above expression. Carbon absorbed by the ocean is not specifically included in this study (likewise by Hammond & Seth, 2013). An equivalence factor for forests of 1.4 gha ha^−1^ was adopted from Alderson *et al*. (2012), and world carbon absorption factor was taken as 0.95 tC ha^−1^ (after Hammond & Seth, 2013). As a result, a conversion factor of 1.017 gha ha^−1^ for the carbon component was obtained. Finally, these numbers were then be multiplied by the IEA estimated global biofuel production to quantify the aggregate carbon component. A similar study by Simmons *et al*. (2000) found that the equivalence factor for forest was 1.14 gha ha^−1^, and a very similar number was subsequently employed by Chambers *et al*. (2000).

To estimate the carbon footprints associated with global biofuel production, the data set on life cycle CO_2e_ emissions for different feedstocks was adopted from an environmental *Life cycle Assessment* (LCA) study sponsored by the UK Government's *Department for Environment, Food and Rural Affairs* (Defra, 2012). [A discussion of the relationship between EFA and LCA can be found in the Supporting information.] The various components were then aggregated to determine the overall footprint of the world biofuel production from the IEA transport roadmap projections (IEA, 2011). The Defra (2012) LCA study reported both direct and indirect biofuel life cycle emission factors, including the entire fuel and end use life cycle known as the ‘well‐to‐wheel’ basis (although it is strictly ‘seed‐to‐wheel’ in terms of energy crops): see Table 2.

**Table 2:** GHG (CO_2e_) emissions from the different feedstocks

Fuel type	RTFO indirect life cycle	Direct CH_4_	Direct N_2_O	Actual life cycle	Direct CO_2_	Total GHG CO_2e_
	Unit (g CO_2_e MJ^−1^)					
Bioethanol	39	0.094	0.172	38.902	71.600	110.502
Bioethanol cane	24	0.094	0.172	24.266	71.600	95.866
Bioethanol SRC	16	0.094	0.172	16.266	71.600	87.866
Biodiesel	34	0.025	0.503	34.182	75.300	109.482
Advanced Biodiesel	23	0.025	0.503	23.528	75.300	98.828
Biomethane	27	0.075	0.031	27.106	55.408	82.514

*Sources*: Both direct and indirect emissions reported by Defra (2012); indirect emissions extracted via DfT (2012) obtained for RTFO purposes.

The indirect emissions under the UK Government's *Renewable Transport Fuel Obligation* (RTFO) are reported by its Department of Transport (DfT, 2012). This RTFO is one of the main UK policies for reducing GHG emissions from road transport. It requires that a certain percentage of transport fuel to be classified as ‘renewable’. Each supplier of fuel to the UK market is therefore required to demonstrate that biofuel has been supplied at a set proportion of their overall fuel supply (this proportion was 4.75% in 2015). In the annual RTFO report, estimates of indirect GHG emissions associated with the fuel production and refining, transport of primary fuels, distribution, storage and retail of finished fuels (although not those from the direct emissions of CO_2_, CH_4_ and N_2_O that are released by combusting biofuels in vehicles). Direct emissions are reported separately by Defra (2012), including those on a ‘well‐to‐tank’ (again strictly ‘seed‐to‐tank’ or ‘plant‐to‐pump’) basis. However, it does provide detailed GHG emissions data associated with the fuel production and actual amount of CO_2_ from burning biofuels [extracted from DfT (2012)]. This was considered to be equal to the amount of CO_2_ sequestered in the growth of the feedstock used to produce the biofuel. CH_4_ and N_2_O are not offset by adsorption in growth of feedstocks, unlike the CO_2_. The total life cycle CO_2e_ emissions employed in this study were obtained by summing the direct emissions of CH_4_ and N_2_O, together with indirect life cycle emissions as reported under the RTFO (DfT, 2012).

#### Embodied energy

The energy embodied in structural materials used for the construction of each biofuel production plant (or biorefinery), along with the operational energy (heat or power) used in the facility, is termed ‘embodied energy’ (Hammond & Jones, 2008). The embodied energy footprint per litre of biofuel worldwide was then calculated (Alderson *et al*., 2012) via:

$$\begin{array}{cc} & \text{Embodied energy footprint component (gha per litre of biofuel)}\\ & \;=\text{Embodied energy (GJ per litre of biofuel)}\\ & \;\times \text{Conversion factor (gha per GJ)} \end{array}$$

The input embodied energy to a biorefinery employed in this study was assumed to be the same amount as the energy required in the fossil fuels industry. The conversion factors were hence computed from primary energy sources and the conversion factors adopted by Alderson *et al*. (2012) (see Table 3). These conversion factors had already taken account of equivalence factors for different land types, which were presented in terms of global hectares per GJ of biofuel. Finally, the results were then multiplied by the IEA estimates of world biofuel production (IEA, 2011) to determine the magnitude of the embodied energy component.

**Table 3:** Embodied energy footprint conversion factors associated with primary and secondary carriers

Energy source	Factors (gha GJ^−1^)
Grid electricity	0.038
Solid fuel	0.023
Petroleum	0.019
Total	0.080
Conversion factor (gha GJ^−1^)	0.027

*Source*: Alderson *et al*. (2012).

#### Transport

The transport component includes the transport of fuel for input into the biorefinery process and onward to the refuelling plant. In principle, it also included the removal of waste products to disposal sites. Thus, the transport footprint per litre of biofuel is estimated as follows (Alderson *et al*., 2012):

$$\begin{array}{cc} & \text{Transport footprint component (gha per litre of biofuel)}\\ & \;=\text{Fuel input (t per litre of biofuel)}\\ & \;\times \text{Conversion factor (gha per t)} \end{array}.$$

Here, the conversion factor was calculated for each mode of transport (based on carbon emissions) and summed as follows (Hammond & Seth, 2013):

$$\begin{array}{cc} & \text{Conversion factor (gha per tC)}\\ & =\sum [\text{Average distance (km)}\times \text{Carbon emissions (tC per t‐km)}\\ & \times \text{Factor (gha per tC)]} \end{array}$$

$$\begin{array}{cc} & \text{Factor (gha per tC)}=\frac{\text{Carbon responsibility}\times \text{Uplift factor}\times \text{Equivalence factor}\;(\text{gha}\;{\text{ha}}^{-1})}{\text{World carbon absorption factor}\;(\text{tC}\;{\text{ha}}^{-1})} \end{array}.$$

The values for the parameters termed carbon responsibility, equivalence factor and world carbon absorption factor adopted here were 0.69, 1.4 gha  ha^−1^ and 0.95 tC  ha^−1^, respectively, for the carbon footprint calculation, after Alderson *et al*. (2012). An ‘uplift factor’ was used to account for the energy which is consumed during the manufacture and maintenance of vehicles for freight purpose, and the necessary infrastructure for road, rail and water. It was assumed that vehicle manufacture and maintenance gives rise to an uplift in carbon emissions of 15%, and that the development of necessary infrastructure added a further 30% to carbon emissions (Alderson *et al*., 2012). Therefore, a total uplift factor of 1.45 was allocated to road, rail and waterborne transport. This was used in the resulting conversion factor to obtain a figure of 1.4744 gha per tC (Alderson *et al*., 2012). The average travelling distance and the associated GHG emissions vary with road, rail and waterborne transport mode (see Alderson *et al*., 2012). Therefore, the conversion factors for each transport mode were calculated separately and then combined to yield an overall conversion factor.

#### Waste arisings

The waste footprint component includes all wastes produced as a result of releases from each biorefinery process, and its footprint is calculated as follows (Alderson *et al*., 2012):

$$\begin{array}{cc} & \text{Waste footprint component (gha per litre of biofuel)}=\frac{\text{Waste arisings (t per litre of biofuel)}\times \text{Equivalence factor}\;(\text{gha}\;{\text{ha}}^{-1})}{\text{World Average Yield}\;(t\;{\text{ha}}^{-1})} \end{array}$$

This equation was used to estimate the waste footprint per litre of global biofuel production. It was then multiplied by the estimated worldwide biofuel production projected by the IEA (2011) to obtain the waste footprint of global biofuels. The method of waste disposal was considered to be landfill only in this study. It was assumed that waste disposal takes up fertile land, which could be otherwise used for agricultural purposes, and therefore, the crop land equivalence factor of 2.1 gha ha^−1^ was employed here (following the practice adopted by Alderson *et al*., 2012). However, the ‘world average yield’ factor for the different types of wastes that are produced during the world biofuel production process varies. The overall waste footprint component was finally computed by multiplying the waste footprint per litre by the IEA projected world biofuel production out to 2050 (IEA, 2011).

#### Water usage

The original ‘water footprint’ adopted by Hoekstra & Hung (2002) provided a framework to analyse the relationship between human activities and global freshwater consumption. The water footprint component per litre of global biofuel production was then computed as follows (Hammond & Seth, 2013):

$$\begin{array}{cc} & \text{Water footprint component (gha per litre of biofuel)}\\ & \;=\text{Consumption of water (litre of water per litre of biofuel)}\\ & \;\times \text{Conversion factor (gha per litres of water)} \end{array}$$

A study of water footprint of crops and derived crop products by Mekonnen & Hoekstra (2011) was employed to determine the global green, blue and grey water requirements for biofuels crop production, although the grey water footprint quantified in the study was solely related to nitrogen use. The conversion factor of 0.102 gha per M litres of water adopted by Alderson *et al*. (2012) was employed to estimate the water footprint component (in terms of gha per litre of biofuel) via the above equation. Finally, the overall water footprint component (gha) was computed by multiplying the water footprint per litre by the IEA projected world biofuel production over the period 2010–2050 (IEA, 2011).

## Results

### Life cycle environmental impact of biofuels

The total life cycle of conventional biodiesel produced 1.11 kgCO_2e_ per litre of biofuel. In total, 50% of these emissions come from the crop plantation stage, whilst the oil extraction and biodiesel production stage contribute around 15% and 28%, respectively (Defra, 2012). By contrast, the conventional bioethanol produced from sugar beet accounted for 0.91 kgCO_2e_ per litre of biofuels. Very similar values were adopted by Delucchi (2006) in his LCA study, who indicated that corn (or maize) bioethanol does not have significantly lower GHG emissions in comparison with petrol (or ‘gasoline’). Indeed, cellulosic bioethanol was found to have only about 50% lower CO_2e_ emissions. The main reason for this (see Hammond & Seth, 2013) is that Delucchi (2006) estimated relatively high CO_2e_ emissions from feedstock and fertilizer production, from land use and cultivation and from emissions of non‐CO_2_ GHGs from vehicles. Therefore, the largest sources for CO_2e_ emissions arose at the upstream end of the biofuels life cycle (Hammond & Seth, 2013); that is those associated with fuel production, feedstock recovery, fertilizer manufacture and ‘displaced’ emissions. Delucchi (2006) observed that the emissions related to feedstock transmission, fuel distribution and liquid‐fuel dispending were relatively small.

### Carbon footprint of biofuels

**Figure 3:**
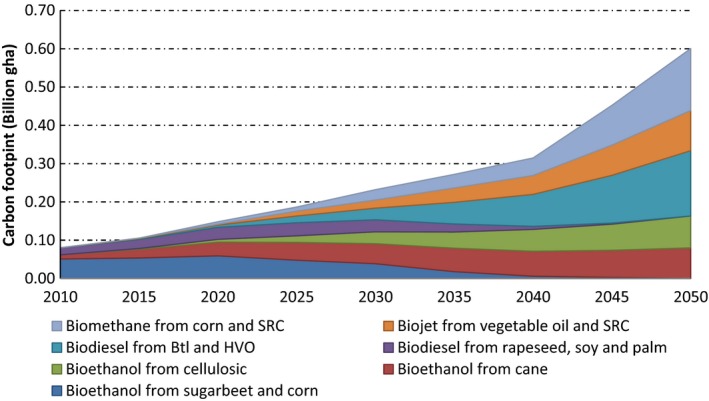
Carbon footprints associated with the world production of various biofuels and feedstocks (2010–2050).

The carbon weight was estimated for each biofuel category from data provided by Defra (2012) for bioethanol, biodiesel and biomethane, respectively. This was then converted into the carbon footprint per tC per litre of biofuel using the conversion factors previously determined (see ‘*Carbon emissions*’ above). The total carbon footprint was obtained from the concatenated results for the individual footprints per litre of bioethanol, biodiesel and biomethane multiplied by the annual IEA biofuel projections out to 2050 (IEA, 2011). It was found to be 0.085 billion (bn) gha in 2010, rising to 0.64 bn gha by 2050 as depicted in Fig. 3. This growth was primarily caused by an increase in bioethanol production from sugarcane and advanced biodiesel. Sugarcane bioethanol produced 0.80 kg CO_2e_ per litre of biofuels, whereas advanced biofuels were found to produce 1.22 kg CO_2e_ per litre of biofuels. Consequently, sugarcane contributed 18% of the total carbon footprint in 2010 and is expected to exhibit a similar proportion by 2050.

The carbon footprint of conventional bioethanol from sugar beet and corn had a combined value of 0.051 bn gha in 2010 and is likely to rise to 0.059 bn gha by 2020. Subsequently, it will gradually be replaced by advanced bioethanol from cellulosic feedstocks (IEA, 2011). In the period 2020–2050, advanced biofuels were found to give rise to <50% GHG emissions compared with conventional (FGB) ones. Over the corresponding period, the carbon footprint emitted from conventional biodiesel extracted from vegetable oil is 0.018 bn gha in 2010, which was projected to rise to 0.034 bn gha by 2025, and then gradually will be replaced by advanced biodiesel (see again Fig. 3). IEA biofuel projections assumed that 50% of advanced biofuels and biomethane are produced from wastes and residues (IEA, 2011). Thus, conventional biodiesel production accounted for 21% of the biofuel carbon footprints in 2010, which is projected to be completely replaced by advanced biodiesel production (such as biojet) by 2050 when it is likely to reflect around 45% of total carbon emissions. IEA global biomethane projections, indicate that there will be a visible increase after 2020 and then a rapid expansion. The carbon footprint of biomethane is then estimated to be 0.162 bn gha in 2050, which contributes 25% of total global biofuel carbon footprint.

### Bioproductive land footprint of biofuels

**Figure 4:**
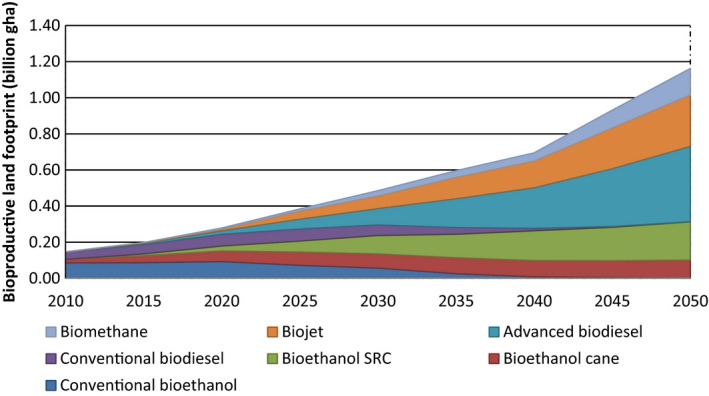
Bioproductive land footprints associated with the world production of various biofuels (2010–2050).

The area of bioproductive land required for each biofuel type was obtained from information provided in the IEA technology roadmap for transport biofuels (2011). These were converted into the bioproductive land footprint per litre of biofuel produced using the conversion factor previously identified (see ‘*Bioproductive land*’ and ‘*Built land*’ above). The bioproductive land footprint component was then calculated for each year from 2010 to 2050 by multiplying the footprint per litre by the projected world biofuel production (IEA, 2011). Bioethanol from sugarcane exhibits a high productivity of 3400 l equivalent per hectare (ha) in 2010, in comparison with conventional bioethanol (2300 l ha^−1^) and conventional biodiesel (2000 l ha^−1^). These accounted for 13%, 58% and 28% of the total productive land footprint in 2010, respectively, due to the larger land productivity and less land area required for unit biofuel produced. The bioproductive land footprints for various biofuel types over the period 2010–2050 are depicted in Fig. 4.

The bioproductive land footprint of conventional biodiesel in 2010 was found to be 0.042 bn gha in 2010, increasing to 0.061 bn gha in 2025 (see again Fig. 4). According to the IEA transport biofuels roadmap (IEA, 2011), this biodiesel will then be gradually replaced by advanced biodiesel (including biojet) from waste and reside during 2025–2050. The latter yields a bioproductive land footprint of 0.418 bn gha by 2050, which amounts to 60% of overall land footprint in that year. In contrast, conventional bioethanol leads to 0.085 bn gha, which accounted for 58% of overall productive land footprint in 2010. This biofuel will be completely replaced by advanced biofuel produced from sugarcane and SRC by 2050 (IEA, 2011), which amounts to 28% of the overall bioproductive land footprint on that timescale.

The total bioproductive land footprint of world biofuel production is also shown in Fig. 4. It amounted to 0.15 bn gha in 2010, but is likely to rise in line with IEA biofuel projections (IEA, 2011) to 1.16 bn gha by 2050. The overall environmental footprint accounts for about a 50% share over the period 2010–2050. To reduce this, large impact of bioproductive land will require improvements in sustainable land management practices, such as greater land productivity, reliance on unused arable land and agriculture intensification. These are methods that might effectively avoid competition between food and fuel crops, as well as reducing the GHG emissions from LUC (IEA, 2010a).

### Water footprint of biofuels

Most water consumption occurs during the agricultural activities which produce the biofuel feedstocks. This water demand was converted into the corresponding water footprint per litre of biofuel produced using the conversion factor previously identified (see ‘*Water usage*’ above). The conversion factor of 0.102 gha per M litres of water adopted by Alderson *et al*. (2012) was employed to estimate the water footprint component (in terms of gha per litre of biofuel). Finally, the overall water footprint component (gha) was computed by multiplying the water footprint per litre by the IEA projected world biofuel production over the period 2010–2050 (IEA, 2011). The initial baseline water footprint was found to be 0.0281 bn gha in 2010.

The water footprint for different crops consists of blue, green and grey water contributions. Conventional biodiesel had a larger water footprint than bioethanol per unit of energy derived. Biodiesel required 7665 l per litre of biofuel, with the green water category accounting for 90% of total water consumption. The corresponding blue and grey footprints each contributed just 5%, respectively. Conventional bioethanol, on the other hand, required 2020 l per litre of biofuel, of which green, blue and grey water contributed 56%, 13% and 31%, respectively. A similar finding was obtained by Gerbens‐Leenes *et al*. (2009). In contrast, advanced biofuels were found to consume only half the water resources of conventional biofuels. This was because the 50% feedstocks were presumed (IEA, 2011) to be derived from waste and residues. A relative high grey water footprint occurs when a large amount of fertilizer is required on the crop field, because it is needed to assimilate nutrients and maintain the water quality. Mekonnen & Hoekstra (2011) found that nutrients leached from agricultural plantations are the main pollution sources that give rise to contamination of the surface and underground water resources.

**Figure 5:**
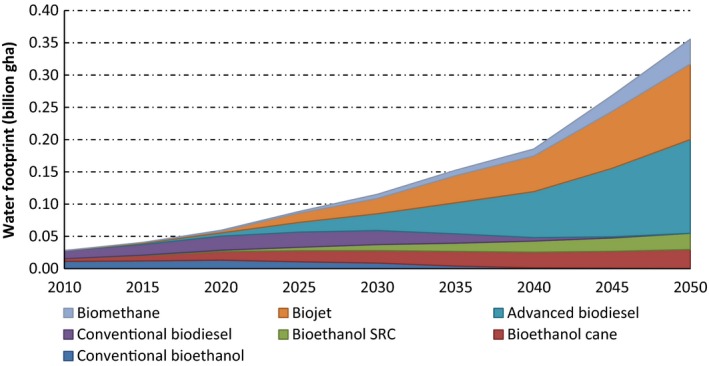
Water footprints associated with the world production of various biofuels (2010–2050).

The overall water footprint was obtained from individual water footprints of the various biofuels. The water footprints for these different biofuels are depicted in Fig. 5 over the period 2010–2050. The water footprint of conventional bioethanol (from sugar beet and corn) was found to be 0.0131 bn gha in 2010, contributing 40% of total water footprint in that year. By contrast, conventional biodiesel from vegetable oil produced 0.0127 bn gha, which accounted 45% of overall water footprint in 2010. The latter will be completely replaced by advanced biofuels by 2050, which contributed 70% of overall water footprint.

The overall water footprint of global biofuel production was 0.028 bn gha in the 2010 baseline year, but is likely to rise to 0.356 bn gha by 2050 (see again Fig. 5). This footprint roughly doubles over the intervening 40 years, although it only accounted for around 9% of total environmental footprint in 2050. Effective ways to cut down the biofuel water footprint on a global scale include encouraging the development of advanced biofuel technologies that yield biofuels from wastes and residues, planting crops that require a minimal amount of fertilizer, and promoting rain‐fed biofuel production.

### Total environmental footprint of biofuels

The overall environmental footprint of global biofuel production for IEA biofuel projection (2011) can be summed in terms of all the individual components. These individual environmental footprint components associated with world biofuel production have been estimated on an annual basis (Hammond & Seth, 2013):

$$\begin{array}{cc} & \text{Total environmental footprint}\;(\text{EF})\\ & \;=\text{Bioproductive land footprint}+\text{Built land footprint}\\ & \;+\text{Carbon footprint}+\text{Embodied energy footprint}\\ & \;+\text{Transport footprint}+\text{Waste footprint}\\ & \;+\text{Water footprint} \end{array}$$

**Figure 6:**
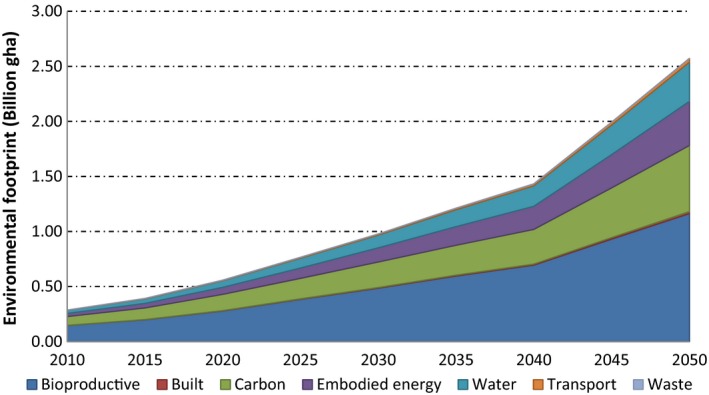
Total environmental footprints associated with world biofuel production and its component shares (2010–2050).

The entire estimation process was then duplicated for each year of the study period, and hence, calculations are best carried out through spreadsheet implementation. The total environmental footprint from different biofuels over the corresponding period is depicted in Fig. 6 below.

The total global biofuel production environmental footprint was estimated to be 0.29 bn gha for 2010 and is likely to grow in line with the IEA transport roadmap projections (IEA, 2011) to 2.57 bn gha by 2050 (see again Fig. 6). Bioproductive land is seen to rise from 0.147 bn gha in 2010 to 1.162 bn gha in 2050. This is proved to be largest footprint component, followed by the carbon footprint component that rose from 0.08 bn gha in 2010 to 0.60 bn gha in 2050, embodied energy from 0.029 bn gha in 2010 to 0.401 bn gha by 2050 and finally water footprint from 0.028 bn gha to 0.356 bn gha by 2050. Thus, bioproductive land and carbon components have contributed around 50% and 25%, respectively, to the overall environmental footprint, whereas embodied energy and water each accounted for roughly 10%, respectively. The footprints of built land, transport and waste were found to account for an insignificant amount to the overall footprint of world biofuel production.

## Discussion

### Biofuel footprints on the landscape

Environmental or ‘ecological’ footprints (ef) have been employed to determine the impacts associated with world biofuel production out to 2050 projected by the IEA as part of their technology roadmap for transport biofuels (IEA, 2011). Such metrics have been widely used in recent years as indicators of resource consumption and waste absorption transformed on the basis of biologically productive land area [in global hectares (gha)] required *per capita* with prevailing technology. They provide a systems integration framework for global sustainability assessment (of the kind advocated by Liu *et al*., 2015) and represent a partial measure of the extent to which an activity is ‘sustainable’. Methodologies employed were consistent with those developed by the GFN and related bodies. In contrast, ‘carbon footprints’ are the amount of carbon [or carbon dioxide equivalent] emissions associated with such activities in units of mass or weight (like kilograms per functional unit), but can be translated into a component of the environmental footprint (on a gha basis). Consequently, *e_f_* has been broken down into different components: bioproductive land, built land, water, carbon emissions (effectively cf), embodied energy, transport and waste components, respectively. This component‐based approach (following Simmons *et al*., 2000; Eaton *et al*., 2007; Alderson *et al*., 2012) facilitates the examination of sustainability issues broadly, along with specific matters, such as the linkages associated with the so‐called ELW nexus (Brandi *et al*., 2013). It provides a means of comparing the various footprint components on a common basis. The approach is not without potential controversy, but yields a better way of comparing environmental burdens than many of the alternatives. These studies represent ‘indicative’ ways of evaluating the performance of world biofuel projections in the light of imperfect information. Such assessments provide a valuable evidence base for developers, policymakers and other stakeholders.

The total environmental footprint of global biofuel consumption was estimated here to be 0.29 bn gha in the base year of 2010, rising to 2.57 bn gha by 2050 (see Fig. 6), based on the IEA projection of world biofuel take‐up [see Table 1 (IEA, 2011)]. Current biofuels are essentially FGB produced primarily from food crops (Hammond *et al*., 2012). They are limited by their inability to achieve targets for oil‐product substitution, without threatening food supplies and biodiversity, and for GHG reductions. Bioproductive land proved to give rise to the highest component of the overall footprint, rising from 0.147 bn gha in 2010 to 1.162 bn gha in 2050 (see also Fig. 4). This distinguishes the footprint results for biofuels from those with other energy sources, such as electricity generation (Alderson *et al*., 2012; Hammond & Seth, 2013), where the land component is relatively small. The carbon footprint of global biofuel production was the next highest [0.080 bn gha in 2010 to 0.600 bn gha in 2050 (see also Fig. 3)], followed by embodied energy (0.029 bn gha in 2010 to 0.401 bn gha in 2050), and then the water footprint [0.028 bn gha in 2010 to 0.07 bn gha in 2019 (see also Fig. 5)]. The built land, transport and waste components contributed an insignificant amount to the total environmental footprint. In order to reduce these impacts, it will be necessary to move towards more advanced biofuels (SGB) produced from agricultural or crop ‘wastes’ (such as straw) and from nonfood energy crops, which reduce these negative environmental burdens (Fairley, 2011; Hammond *et al*., 2012; Hammond & Seth, 2013).

### The implications of the ‘energy–land–water nexus’

The term ‘natural capital’ is typically used to denote the stock of natural assets and resources that yield ecosystem goods and services, such as those required for food (including those associated with pollination of crops), timber and the absorption or recycling of human waste arisings (together with CO_2_), as well as water catchment and erosion control. Maintenance of this natural capital is consequently central to securing environmental security and sustainability over the longer term. In turn, a key subset is the so‐called nexus, or set of complex interactions, between energy requirements, land uses and water consumption levels worldwide. This ELW nexus (Brandi *et al*., 2013) gives rise to multiple positive and negative impacts that have become widely recognized in policy making circles. The generation of energy vectors is obviously the main driver for anthropogenic climate change, whilst there are competing demands on land use [both LUC and iLUC (Hammond *et al*., 2012; Hammond & Seth, 2013)] for both food and biofuel production. Water is needed for drinking, irrigation, food and biofuel crop production, hydro‐electric dams and various leisure pursuits. They are all exacerbated by increasing ELW demands arising from the growth in world population that is moving towards 8 bn in 2025 and 9.5 bn by 2050 (Cranston & Hammond, 2010), as well as human socio‐economic developments generally. Such demands are often framed in terms of energy, food or water ‘security’. It is argued that a strategy which focuses on just one element of the nexus, without considering the others, is likely to lead to major unintended consequences. Thus, many have advocated the need for an integrated approach to the management and governance of nexus issues across various sectors and at different scales to ensure sustainability. This would necessitate research and the modelling of ELW impacts within an informed, transparent and integrated framework for planning and decision support.

Environmental footprinting provides an, albeit imperfect, approach to evaluating natural capital or ecosystem services impacts that arise from the ELW demands of humanity (Brandi *et al*., 2013). An estimate of the global amount of water required per litre of biofuel production was computed here for the overall life cycle of global biofuel production, which is mainly used during the agricultural activities that produce the biofuel feedstocks. These were employed to calculate the water footprint per litre of biofuel produced (see ‘*Water usage*’ and ‘*Water footprint of biofuels*’ above). The IEA projection of global biofuel production (IEA, 2011), and conversion (or ‘equivalence’) factors, was then used to determine the water footprint in global hectares for each year from 2010 to 2050. Different crops were considered, along with their blue, green and grey water requirements. The total water footprint for global biofuel production was found to be 0.0281 bn gha in 2010, rising to 0.356 bn gha by 2050 (see Fig. 5). It doubled over these 40 years and will account for around 9% of total environmental footprint in 2050. [But it should be borne in mind that, on the basis of the methodology employed here, significantly higher contributions emanated from bioproductive land use and carbon emissions (48% and 23%, respectively).] Nevertheless, advanced (SGB) biofuels (Hammond *et al*., 2012; Hammond & Seth, 2013) only resulted in about half the water footprint of FGB, because it was assumed that 50% of their feedstocks were obtained from waste and residues. A relatively greater grey water footprint was observed due to the significant use of fertilizer required in cultivation of those crops. This resulted in a large amount of ‘grey water’ being needed to dilute nutrient concentrations that leach from agricultural plantations and thereby to maintain water quality. Hoekstra & Hung (2002) observed, for instance, that such nutrients are the principal contaminant sources giving rise to the pollution of surface and underground water. Thus, encouragement of the take‐up of advanced biofuels from wastes and residues, the planting of crops that require only a minimal amount of fertilizer or the promotion of rain‐fed biofuel feedstocks are all likely to be effective ways of reducing the water footprint associated with world biofuel production out to 2050.

## Supporting information


**Appendix S1**. Electronic supplementary information.
