# Supplementary material for: Environmental and resource burdens associated with world biofuel production out to 2050: footprint components from carbon emissions and land use to waste arisings and water consumption
Source: Glob Change Biol Bioenergy. 2016 Jan 5;8(5):894–908. doi: 10.1111/gcbb.12300 (PMC4988122; doi:10.1111/gcbb.12300)
Supplement: Supplementary file 1 — Appendix S1. Electronic supplementary information. [file GCBB-8-894-s001.doc]

**Electronic Supplementary Information (ESI) *for* “Environmental and resource burdens associated with world biofuel production out to 2050: Footprint components from carbon emissions and land use to waste arisings and water consumption”**

Geoffrey P. HAMMOND^a,b^ and LI Bo^a^

^a^ *Department of Mechanical Engineering, University of Bath, Bath.BA2 7AY.United Kingdom.*

^b^ *Institute for Sustainable Energy and the Environment (I•SEE), University of Bath, Bath.BA2 7AY.United Kingdom.*

The following material provides an additional background narrative description of various biofuels and feedstocks, a discussion of relationship between *Environmental Footprint Analysis* (EFA) and Environmental *Life-cycle Assessment* (LCA), comparisons with the results of the earlier study of global biofuel footprints out to 2019 by Hammond & Seth (2013), and associated literature references the supplementary text.

**Biofuels, feedstocks, and upstream impacts**

Biofuels are fuels which have been produced from renewable resources, such as plant biomass, vegetable oils, and treated municipal and industrial wastes (Tester *et al.*, 2005; Hammond & Seth, 2013). They are considered potentially ‘carbon neutral’, due to the fact that the plants absorb the carbon dioxide which is released when they are burnt. The extent to which CO_2_ emissions are actually reduced depends on (direct and indirect) land use changes associated with their production. Thus, the life-cycle GHG savings associated with various types of biofuel range from about + 85% to -30%, depending on the feedstock involved and their production methods (Delucchi, 2006). The use of biofuels as an additive to petroleum-based fuels (with low or high blends) can also result in cleaner burning with smaller releases of carbon monoxide and particulates (Tester *et al.*, 2005). In addition to climate change mitigation, biofuels help to diversify and improve security of energy supplies, as well as aiding rural development (Hammond *et al.*, 2012).

Bioethanol is currently produced using first generation technology (i.e., FGB) to ferment and then distil sugar (e.g., from sugarcane, sugar beet, and sweet sorghum) or starchy crops (e.g., corn, wheat, and cassava). It is therefore typically produced by fermentation of crops high in sugar, or by a series of hydrolysis/fermentation steps for starchy materials (Hammond *et al.*, 2008). Bioethanol has long been produced from sugarcane in Brazil, and from corn (maize) and soybeans in the USA (Elghali *et al.*, 2007). It is viewed as a cleaner-burning replacement for petroleum-based oil. However, the energy requirements for starch-based bioethanol are significantly greater than that for sugar-based bioethanol, due to the process of first converting starches into sugar. Bioethanol production from sugar crops is consequently preferred on performance grounds over starchy crops as their energy and GHG balances are preferable (Hammond *et al.*, 2008). In contrast, biodiesel is a liquid fuel made up of fatty acid alkyl esters, fatty acid methyl esters (FAME), or long-chain mono alkyl esters. It is [produced](http://www.afdc.energy.gov/afdc/fuels/biodiesel_production.html) from either oil extracted from seeds or oil-rich nuts, or recovered waste vegetable oils and animal fats (Hammond *et al.*, 2008; Hammond & Seth, 2013). Biodiesel is obtained by transesterification of these feedstocks to produce methyl ether (Hammond *et al.*, 2008). Such biodiesel can be used in compression-ignition diesel engines, normally as a 5% blend, although it can be employed at 100% in specially-modified engines (Hammond *et al.*, 2008).

The next (second and third) generation biofuel technologies are considered to offer a potential solution for some of the sustainability issues associated with FGB (Hammond *et al.*, 2012; Hammond & Jones, 2011). SGB are based on cellulosic biomass, which includes herbaceous lignocellulosic species [such as miscanthus, switchgrass and reed canary grass (perennial crops) and trees such as poplar, willow and eucalyptus (short rotation crops)], as well as forestry and agricultural residue (Hammond *et al.*, 2008; Hammond & Jones, 2011). Biodiesel is derived from various agricultural products depending on their availability in different regions. In India, [Jatropha](http://altenergy.in/jatrophacurcas.html) and Pongamia (a genus of legume in the *Fabaceae* family) are presently used to produce biodiesel (Hammond & Seth, 2013). Palm oil is in widespread production in South East Asia to generate a high yield biofuel, which is an edible (albeit relatively expensive) advanced feedstock option for the longer term (and it is therefore often referred to as a ‘third generation’ biofuel). Algae are also being evaluated and demonstrated as a diesel fuel and related co-products (Hammond & Seth, 2013).

SGB can reduce life-cycle GHG emissions, because of their higher energy yields per hectare and the potential of remaining plant material (mostly lignin) that can be employed as process energy. The greatest sources of emissions are in the upstream stage of the biofuel life-cycle (Delucchi, 2006). These include land-use changes and cultivation, fuel production, feedstock recovery, fertilizer manufacture, and ‘displaced’ emissions [sometimes referred to, perhaps inappropriately, as ‘co-product credits’ (Delucchi, 2006)]. However, the conversion technologies are at a relatively early stage of development (Hammond *et al.*, 2008). Substantial technological and economic barriers impede their commercial deployment (Adams *et al.*, 2011; Hammond *et al.*, 2012), including high production costs, logistics, and supply chain challenges. Another important barrier is the set of agricultural/forestry sector practices needed to regularly supply the lignocellulosic feedstock. These depend on changes in agricultural management, as well as policy changes, both of which will take time to implement (Adams *et al.*, 2011).

**The Relationship between Environmental Footprint Analysis (EFA) and Environmental Life-cycle Assessment (LCA)**

The energy analysis and environmental appraisal of bio-based products and energy systems ideally need to be conducted on a life-cycle basis, i.e., embracing the full range of extraction, production, distribution, and end-of-life processes or technologies (Reap *et al.*, 2008; Hammond *et al.*, 2015). This approach involves what is now known as environmental life-cycle assessment (LCA); codified as *International Standards Organization* (ISO) 14040 series of standards (ISO, 2006a; ISO, 2006b). The aim of an LCA study is often to identify opportunities for environmental improvement by detecting the areas with the most significant impacts. In a comprehensive, ‘full’ or ‘detailed’ LCA, the energy and materials used, and pollutants or wastes released into the environment as a consequence of a product or activity are quantified over the whole life-cycle, ‘from cradle-to-grave’ (Baumann & Tillman, 2004; Vogtländer, 2010; Curran, 2012; Hammond *et al.*, 2015). The current strengths and weaknesses of LCA have recently been identified by Hammond *et al.* (2015) for the benefit of users, particularly energy practitioners and policy analysts: see Table S1. A similar range of advantages and disadvantages were also identified by Čuček *et al.* (2012) in their review aimed at evaluating various tools for monitoring sustainability impacts.

**Table S1: An Outline of the Strengths and Weaknesses of Environmental LCA.**

| **Strengths** | **Weaknesses** |
| --- | --- |
| Holistic environmental appraisal | Static/Snapshot assessments |
| Established international standards | Variation in assessment due to value choice/ methodological approaches |
| Procedural transparency | Only predefined environmental impacts assessed |
| Allows level playing field for comparison | A target for sustainable activity not specified only embodied impacts quantified |
| Pinpoints environmental/inefficient hotspots | Data quality |
| Springboard for communication | Inaccessible results |

*Source:* Hammond *et al.* (2015).

There has been an increasing interest amongst researchers and practitioners in the relationship and interaction between EFA and LCA (Huijbregts *et al.*, 2008; Castellani & Sala, 2012; Hammond & Seth, 2013). Čuček *et al.* (2012) recently compared and contrasted several different footprints (e.g., environmental, social, economic and composite metrics), and highlighted their links with LCA. Another particularly useful comparison between the EFA and LCA methods was reported by Castellani & Sala (2012) in the context of a sustainability assessment of tourism activities in Italy. They drew out the main comparative strengths and weaknesses of the EFA and LCA approaches. The former does not capture the full range of environmental impact categories (Castellani & Sala, 2012) embracing, for example, damage to resources (resulting from the consumption of fossil fuels and other minerals), damage to ecosystem quality (caused by acidification, eutrophication, ecotoxicity, etc.), and damage to human health (due to human toxicity). On the other hand, EFA provides a useful means of environmental monitoring against a specific physical threshold: the amount of land available. Unlike *Life Cycle Impact Assessment* (LCIA), EFA also takes account of limited natural resources or the carrying capacity of the planet (Huijbregts *et al.*, 2008). However, the EFA approach doesn’t allow for the multi-purpose use of ecosystems, e.g., to sustain biodiversity, for timber production, and for carbon sequestration. Castellani & Sala (2012) explore the interactions between footprint components, like the seven different components used in the present work, and typical LCA impact categories and associated inventory data. They note that collecting primary data from specific LCA studies of each consumption category will enhance the robustness of EFA. Another recent study by Huijbregts *et al.* (2008) again examined the interrelation between EFA and LCA, but for a range of some 1550 product/process groups consumed in the industrialised global economy. They used the Eco-indicator 99 (EI) LCIA method (Goedkoop & Spriensma, 2001), and found that the EF/EI ratio was constant to within a variation of about ±17%. Considerations of this type have led leading EFA practitioners to place the acquisition of better data sources (including those from LCA studies) high on their research agenda (Kitzes *et al.*, 2009).

**Discussion of the Calculation of Biofuel Footprints**

The current study has employed the world biofuel projections developed for the IEA transport roadmap (IEA, 2011), which covered seven categories of biofuels produced from different feedstocks and technologies. They included the conventional bioethanol produced from sugar and starch crops, advanced bioethanol produced from cellulosic feedstocks, conventional biodiesel derived from vegetable oil, advanced biodiesel from ‘hydrotreated vegetable oil’ (HVO) and ‘biomass to liquid’ (BtL) technology, and biomethane. Although the IEA projection covered a wide range of conventional and advanced biofuel conversion technologies, only conventional biofuel technologies are commercially and relatively mature already. Most of advanced biofuel approaches currently are still in a critical stage of technology development which requires reaching a commercial scale and being widely deployed. Therefore, the potential uncertainty of biofuel yield and conversion efficiency over long-term is still relatively large, especially over the next 40 years.

The collection of the primary consumption data for variety footprint components as well as the energy and source flow into and out of the biofuel production sector are required at the early stage of footprint analysis. Some secondary data adapted from international statistics was employed in the absence of sector-specific primary data. These data are normally provided by individuals or organizations, which are considered either confidential or vague that do not have sufficient credible details. Hammond & Seth (2013) consequently noted that the provision of better primary and secondary data sources would enhance the robustness of EFA and improve the confidence with which the sustainability assessment is viewed. Indeed, Kitzes *et al.* (2009) concluded that the acquisition of better data sources (including those from LCA studies) should be placed high on the research agenda of the EFA research and practitioner communities.

EFA has been widely used to evaluate the extent to which human activities are sustainable. Hammond & Seth (2013) recently observed that bioenergy and biofuel footprints and land-take reflect relatively large environmental burdens when compared to other fuels. Unfortunately, the environmental impacts associated with the bulk expansion of the world biofuel market have scarcely been addressed. Factors such as the use of nitrogen fertiliser during cultivation activities, co-product allocation, and the deterioration in global water resources and their quality are often uncertain. Moreover, some other poorly understood environmental impacts, including the carbon sequestration throughout the biofuel feedstock growth cycle, may lead to an underestimation of overall footprint and its various components.

## Comparison with Previous Biofuel Footprint Studies

It is informative to compare and contrast the findings of the present study with the earlier one by Hammond & Seth (2013). The latter utilised the global biofuel production estimates reported by the OECD-FAO (2010) for the period 2010-2019. This led to a total environmental footprints that was roughly 2.5 times greater than those obtained in the present study over the timescale to approximately 2020 (see Fig. S1). The Hammond & Seth (2013) calculations found that for the OECD-FAO biofuel projections indicated that the total footprint rose from 0.4 bn gha to 0.67 bn gha over the period 2010-2019; in contrast to those of the present study, based on the IEA transport biofuel roadmap (IEA, 2011), suggest an increase from 0.15bn gha to 0.24bn gha on the same timescale. The contribution of bioproductive land component in the Hammond & Seth (2013) was about 55% to the total environmental footprint (compared with a 45% share in 2050 here), whereas the carbon footprint contributed around 35% (23% here), and water and transport each of accounting for 5% (14% water footprint here, but with an insignificant contribution from transport).


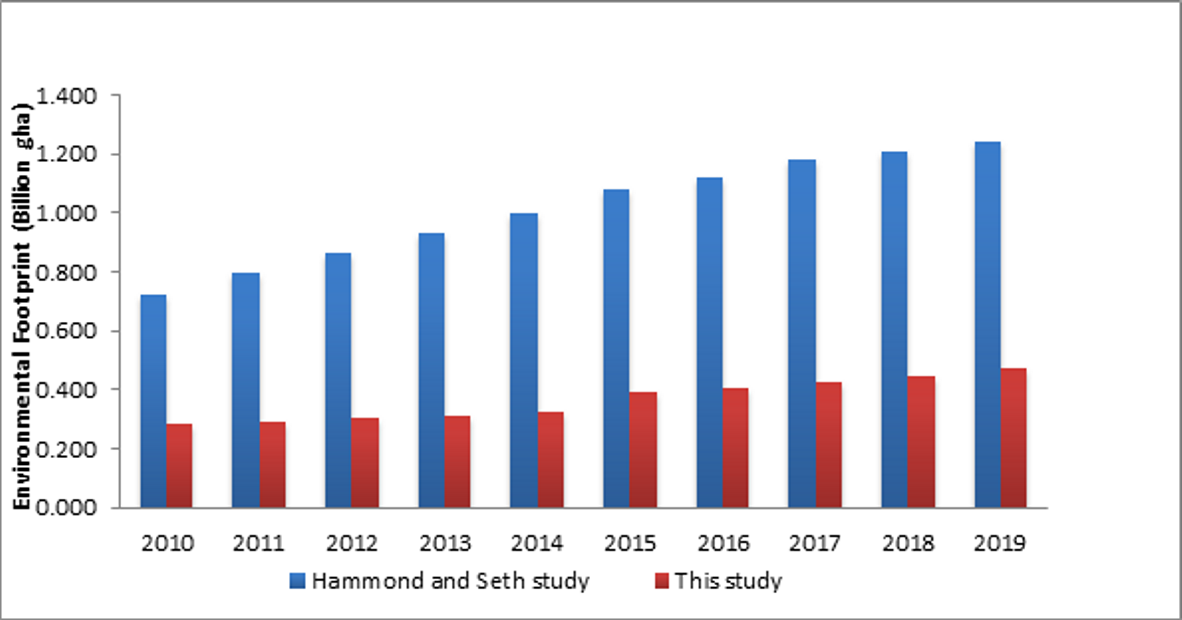


**Fig. S1. Total environmental footprints associated with OECD-FAO and IEA world**

**biofuel projections (2010-2019). [*Sources:* Estimates based on the OECD-FAO**

**biofuel extrapolations (Hammond & Seth, 2013); IEA biofuel roadmap utilised**

**in the present study].**

The carbon emissions (CO_2e_) component reflects the main difference between the studies by Hammond & Seth (2013) and the present one: see Fig. S2. Projections of biofuel demand by OECD-FAO (2010) resulted in a carbon footprint of 0.248bn gha in 2010 rising to 0.449bn gha by 2019. In contrast, the present study using the IEA transport biofuel roadmap (IEA, 2011) displayed a rise from 0.08bn to 0.125bn gha during the same period of study, see again Fig.S2. Hammond & Seth (2013) indicated that the main contribution to their carbon footprint came from conventional biodiesel produced from vegetable oil. It contributed nearly 70% of total carbon emissions component, whereas biodiesel from vegetable oil only contribute 20% over the corresponding period. In contrast, the main GHG source in the


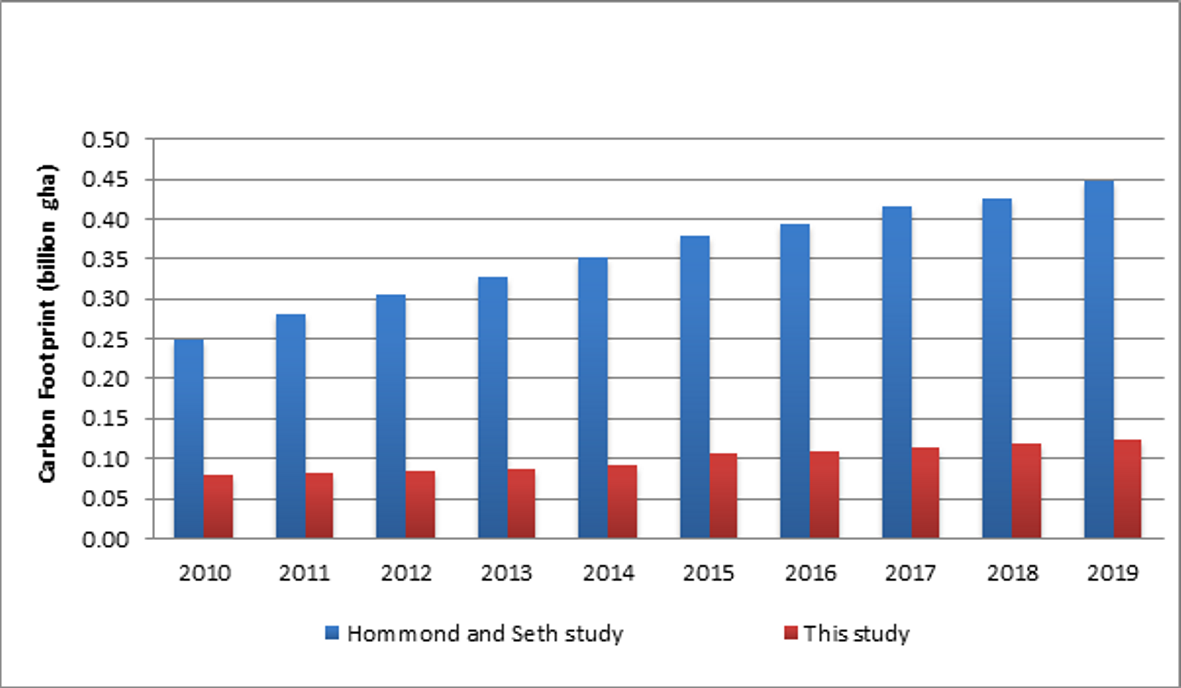


**Fig. S2 Carbon footprints associated with OECD-FAO and IEA world biofuel**

**projections (2010-2019). [*Sources:* Estimates based on the OECD-FAO biofuel**

**extrapolations (Hammond & Seth, 2013); IEA biofuel roadmap utilised in the**

**present study].**

present study was bioethanol produced from combined corn and sugar beet and that from sugarcane (which accounted for 64% and 14% of the total carbon emissions in 2010, increasing to 45% and 26% by 2019). This discrepancy primarily came from the differences of global biofuel projection between the OECD-FAO (2010) and IEA (2011), and the life-cycle CO_2e_ emissions attributable to the variety biofuel categories from the different feedstocks [those for the IEA (2011) roadmap are shown in Table 1 (main paper)].

The conversion factor used in estimation to determine the transport footprint (see ‘*Transport*’ above) by Hammond & Seth (2013) was found to be exaggerated by almost an order of magnitude. A comparison of the transport footprint is shown in Fig. S3. However, this disparity had only a small effect on the overall footprint, since the transport component only accounted for less than 5% of total environmental footprint in both studies. In the current study a conversion factor of 1.474gha/tC was employed [as previously adopted by Alderson *et al.* (2012) for their study of UK power generation futures] to estimate the transport footprint of global biofuel production out to 2050. This footprint may change significantly in the future, as it is clearly desirable to locate conversion technologies close to feedstock sources in order to minimise the need for transportation vehicles and infrastructure. The transport of biofuels at present is primarily by road, rail, and ship, which yields a relatively large environmental footprint and are less cost-effective than via pipeline shipment (WEC, 2011). Some fuel suppliers and producers have shown an interest in building dedicated pipelines, or in the use of existing pipelines, to simplify downstream product distribution.


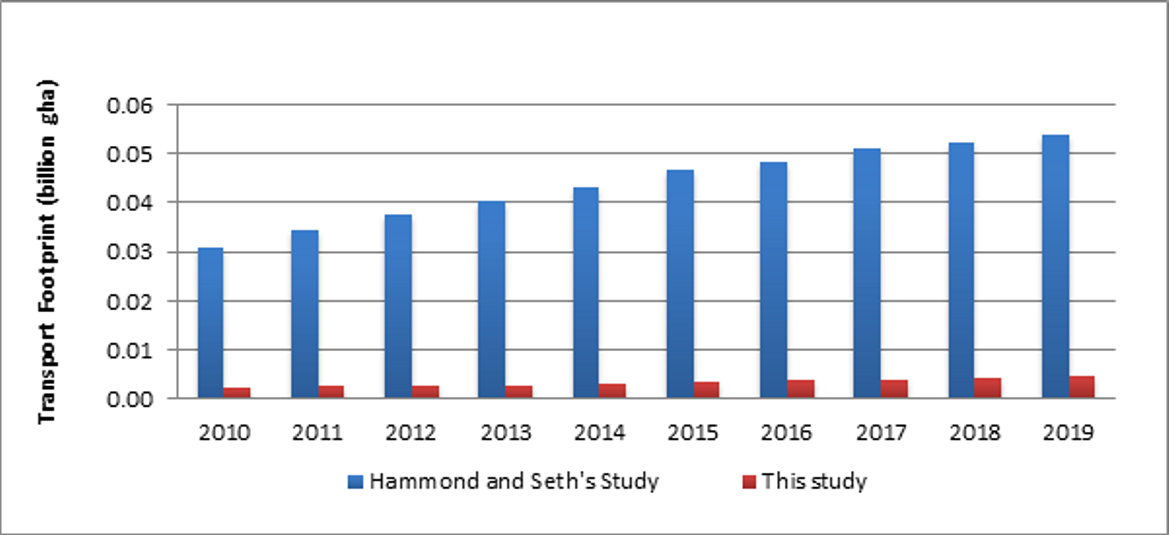


**Fig. S3 Transport footprint components associated with OECD-FAO and IEA world**

**biofuel projections (2010-2019). [*Sources:* Estimates based on the OECD-FAO**

**biofuel extrapolations (Hammond & Seth, 2013); IEA biofuel roadmap utilised**

**in the present study].**

**References to Publications Cited in the Supplement Material**

Adams, PW, Hammond GP, McManus MC, Mezzullo WG (2011) [Barriers to and drivers for UK bioenergy development](http://www.sciencedirect.com/science?_ob=ArticleURL&_udi=B6VMY-514RB5B-3&_user=126089&_coverDate=10%2F01%2F2010&_rdoc=12&_fmt=high&_orig=browse&_origin=browse&_zone=rslt_list_item&_srch=doc-info(%23toc%236163%239999%23999999999%2399999%23FLA%23display%23Articles)&_cdi=6163&_sort=d&_docanchor=&_ct=96&_acct=C000010279&_version=1&_urlVersion=0&_userid=126089&md5=0f8f6d016f582ee07c12fbb00d9720db&searchtype=a). *Renewable and Sustainable Energy Reviews*, **15**(2): 1217-1227.

Alderson H, Cranston GR, Hammond GP (2012) Carbon and environmental footprinting of low carbon UK electricity futures to 2050. *Energy*, **48**(1): 96-107.

Baumann H, Tillman A-M (2004) *The Hitch Hiker's Guide to LCA: An orientation in life cycle assessment methodology and application*, Studentlitteratur, Lund, Sweden.

Castellani V, Sala S (2012) Ecological footprint and life cycle assessment in the sustainability assessment of tourism activities. [*Ecological Indicators*](http://www.sciencedirect.com/science/journal/1470160X), **16**: 135–147.

Čuček L, Klemeš JJ, Kravanja Z (2012) A review of footprint analysis tools for monitoring impacts on sustainability. *Journal of Cleaner Production*, **34**: 9-20.

Curran MA, editors (2012) *Life Cycle Assessment Handbook: A Guide for Environmentally Sustainable Products*, Wiley-Scrivener, Beverly, MA, USA/Chichester, UK.

Delucchi MA (2006) *Lifecycle Analysis of Biofuels*. ReportUCD-ITS-RR-06–08, Institute of Transportation Studies, University of California, Davis, CA.

Elghali L, Clift R, Sinclair P, Panoutsou C, Bauen A (2007) Developing a sustainability framework for the assessment of bioenergy systems. *Energy Policy*, **35**(12): 6075-6083.

Goedkoop M, Spriensma R (2001) *The Eco-indicator 99: A Damage Oriented Method for Life-cycle Impact Assessmen*t, 3^rd^ ed., PRé Consultants, Amersfoort, The Netherlands.

Hammond GP, Howard HR, Tuck A (2012) Risk assessment of UK biofuel developments within rapidly evolving energy and transport sectors. *Proc. Instn Mech. Engrs Part O: Journal of Risk and Reliability*, **226**(5): 526-548.

Hammond GP, Jones CI (2011) Sustainability criteria for energy resources and technologies. In: Galarraga I, González-Eguino M, Markandya A, editors. *The Handbook of Sustainable Energy*, Cheltenham: Edward Elgar; Ch. 2, p. 21-46.

Hammond GP, C.I. Jones CI, O’Grady A (2015) Environmental life cycle assessment (LCA) of energy systems. In: *Handbook of Clean Energy Systems*, *Vol. 6.* Yan J (ed.), John Wiley and Sons, New York, in press.

Hammond GP, Kallu S, McManus MC (2008) The development of biofuels for the UK automotive market. *Applied Energy*, **85**(6): 506-515.

Hammond GP, Seth SM (2013) Carbon and environmental footprinting of global biofuel production. *Applied Energy*, **112**: 547-559.

Huijbregts MAJ, Hellweg S, Frischknecht R, *et al.* (2008) Ecological footprint accounting in the life cycle assessment of products. [*Ecological Economics*](http://www.sciencedirect.com/science/journal/1470160X), **64**: 798–807.

International Energy Agency [IEA] (2011) *Technology Roadmap: Biofuels for Transport,* OCED /IEA, Paris.

International Standards Organization [ISO] (2006a) *Environmental management – life cycle assessment – principles and framework*, EN ISO 14040, 2^nd^ ed., ISO, Geneva.

International Standards Organization [ISO] (2006b) *Environmental management – life cycle assessment – requirements and guidelines*, EN ISO 14044, ISO, Geneva.

Kitzes J, Galli A, Bagliani M, *et al.* (2009) A research agenda for improving national Ecological Footprint accounts. [*Ecological Economics*](http://www.sciencedirect.com/science/journal/1470160X), **68**: 1991–2007.

Organisation of Economic Co-operation and Development [OECD] - Food and Agricultural Organisation [FAO] (2010) *OECD-FAO Agricultural Outlook 2010-2019*, OCED, Paris.

Reap J, Roman F, Duncan S, Bras B (2008) A survey of unresolved problems in life cycle assessment: Part 1 Goal and scope definition and inventory analysis. *The International Journal of Life Cycle Assessment*, **13**(5): 290–300.

Tester JW, Drake EM, Driscoll MJ, Golay MW, Peters WA (2005) *Sustainable Energy: Choosing Among Options*, MIT Press, Cambridge, MA.

Vogtländer JG (2010) *LCA: a Practical Guide for Students, Designers and Business Managers*, VSSD, Delft, The Netherlands.

World Energy Council [WEC] (2011) *Global Transport Scenarios 2050*, WEC, London.
